# Editorial—Pesticide Risk Assessment, Emerging and Re-Emerging Problems

**DOI:** 10.3390/toxics14010008

**Published:** 2025-12-20

**Authors:** Claudio Colosio

**Affiliations:** Post Graduate School in Occupational Health, University of Milano, Via S, Barnaba 8, 20122 Milan, Italy; claudio.colosio@unimi.it

Nearly half of the world’s eight billion people live and work in rural areas, most of them employed in agriculture [1], one of the most hazardous occupations according to the International Labor Organization [2]. Rural populations and workers are exposed daily to multiple occupational and environmental risks, and pesticides represent one of the most critical among them [3]. These compounds are intentionally designed to be toxic and, unlike most chemicals, must be dispersed into the environment to reach their targets. Their toxicity is rarely completely selective for pest species, raising concerns about unintended effects on ecosystems, wildlife, and humans [4].

In this context, pesticide risk assessment remains fundamental, yet increasingly challenged by rapidly evolving pesticide markets, emerging formulations, heterogeneous regulatory capacities, and the persistence of obsolete products in low-income regions. Real-world exposure scenarios are highly variable, and biological or environmental monitoring is often performed only after exposure has already occurred. At the same time, the rapid growth of information technologies is opening new opportunities for modelling tools capable of supporting decision-making in agriculture, although most current models remain focused on pre-marketing evaluations rather than daily operational needs. These considerations frame the scientific contributions gathered in the Special Issue “Pesticide Risk Assessment, Emerging and Re-Emerging Problems.”

Dermal absorption, one of the most relevant routes of human pesticide exposure, is addressed by several contributions. Städele et al. reassessed the default dermal absorption values currently used in European regulatory guidance by analysing a new dataset of 356 human in vitro studies. Their results suggest that updated default values—especially for diluted solid formulations—would be considerably lower than those presently adopted, indicating that a revision of regulatory benchmarks may be warranted (Contribution 1).

Pieper et al. evaluated a dataset of 945 dermal absorption experiments involving 179 active substances in 353 mixtures. Their analysis supports the appropriateness of current default values and the pro-rata correction for untested dilutions, while highlighting the potential influence of specific co-formulants in shaping absorption outcomes (Contribution 2).

Emerging formulations are explored by Rud et al., who investigated the fate and transport of nano-formulated and conventional pesticides in agricultural field plots. Their findings reveal significant effects on nutrient cycling, including changes in ammonium, total nitrogen, and orthophosphate in runoff, alongside persistent copper accumulation in soils. These results underscore the need to incorporate biogeochemical dynamics into nanopesticide risk assessments (Contribution 3).

A broader regulatory and public health perspective is presented by Singh et al., who developed a national chemical prioritisation strategy for human biomonitoring in Ireland. By integrating European and international frameworks with public perception data, the authors identified priority groups such as metals, plasticisers, bisphenols, PFAS, PAHs, and pesticides, offering a robust and adaptable roadmap for evidence-based policy development (Contribution 4).

Rubino et al. describe the development of SICURPEST, a user-friendly software tool designed to support farmers in preliminary pesticide risk assessment prior to application. Built within a research project, SICURPEST funded by the Italian Workers’ Compensation Authority (INAI) integrates exposure determinants and product-specific parameters, providing a simplified model intended for practical use at the farm level (Contribution 5).

Environmental health implications are examined by da Silva Lima et al., who analysed Alzheimer’s disease mortality, life expectancy, socioeconomic indicators, and pesticide sales across Brazil from 2010 to 2020. Their strong positive correlations raise important questions regarding environmental determinants of neurodegenerative disease and point toward areas requiring deeper epidemiological research (Contribution 6).

Analytical chemistry remains central to pesticide regulation. Pacini et al. review methodological advances and persistent limitations in the detection of dithiocarbamates and their degradation products in plant-derived foods. Despite progress, challenges remain concerning environmental sustainability of current methods, inconsistent recovery efficiencies, and limited reproducibility across commodity groups (Contribution 7).

The growing interest in sustainable pest management is represented by Daraban et al., who compare conventional pesticides with biopesticides in terms of toxicity, environmental impact, and potential for resistance. While biopesticides offer meaningful benefits—such as reduced persistence and lower non-target toxicity—their limitations highlight the need for deeper research and regulatory clarity (Contribution 8).

Finally, Moreira et al. systematically review novel biomonitoring techniques for pesticide exposure among agricultural workers. New analytical approaches demonstrate improved sensitivity and specificity and may complement existing qualitative methods, supporting more effective protection of both environmental and occupational health (Contribution 9).

Collectively, the papers in this Special Issue illustrate the multifaceted evolution of pesticide risk assessment. They highlight the scientific, regulatory, and practical challenges posed by emerging technologies, exposure pathways, analytical methods, and public health concerns. As Guest Editor, I extend my sincere appreciation to all authors, reviewers, and the Toxics editorial team for their valuable contributions. I hope this collection will stimulate further dialogue and innovation toward safer and more sustainable pesticide use worldwide.

## Data Availability

No new data were created or analyzed in this study. Data sharing is not applicable to this article.
